# Unrecognized diversity and distribution of soil algae from Maritime Antarctica (Fildes Peninsula, King George Island)

**DOI:** 10.3389/fmicb.2023.1118747

**Published:** 2023-06-26

**Authors:** Nataliya Rybalka, Matthias Blanke, Ana Tzvetkova, Angela Noll, Christian Roos, Jens Boy, Diana Boy, Daniel Nimptsch, Roberto Godoy, Thomas Friedl

**Affiliations:** ^1^Department of Experimental Phycology and Culture Collection of Algae (EPSAG), Albrecht-von-Haller-Institute for Plant Sciences, Georg August University, Göttingen, Germany; ^2^Department of Bioinformatics, Institute of Microbiology and Genetics, Georg August University, Göttingen, Germany; ^3^Institute of Bioinformatics and Human Molecular Genetics Group, Department of Functional Genomics, Interfaculty Institute of Genetics and Functional Genomics, University Medicine Greifswald, Greifswald, Germany; ^4^Primate Genetics Laboratory, German Primate Center, Leibniz Institute for Primate Research, Göttingen, Germany; ^5^Institute of Soil Science, Leibniz University, Hanover, Germany; ^6^Institute of Microbiology, Leibniz University, Hanover, Germany; ^7^Instituto de Ciencias Ambientales y Evolutivas, Universidad Austral de Chile, Valdivia, Chile

**Keywords:** soil algae, green algae, Xanthophyceae, Antarctica, Fildes Peninsula, paired-end (ITS2) sequencing, distribution

## Abstract

**Introduction:**

Eukaryotic algae in the top few centimeters of fellfield soils of ice-free Maritime Antarctica have many important effects on their habitat, such as being significant drivers of organic matter input into the soils and reducing the impact of wind erosion by soil aggregate formation. To better understand the diversity and distribution of Antarctic terrestrial algae, we performed a pilot study on the surface soils of *Meseta*, an ice-free plateau mountain crest of Fildes Peninsula, King George Island, being hardly influenced by the marine realm and anthropogenic disturbances. It is openly exposed to microbial colonization from outside Antarctica and connected to the much harsher and dryer ice-free zones of the continental Antarctic. A temperate reference site under mild land use, *SchF*, was included to further test for the *Meseta* algae distribution in a contrasting environment.

**Methods:**

We employed a paired-end metabarcoding analysis based on amplicons of the highly variable nuclear-encoded ITS2 rDNA region, complemented by a clone library approach. It targeted the four algal classes, Chlorophyceae, Trebouxiophyceae, Ulvophyceae, and Xanthophyceae, representing key groups of cold-adapted soil algae.

**Results:**

A surprisingly high diversity of 830 algal OTUs was revealed, assigned to 58 genera in the four targeted algal classes. Members of the green algal class Trebouxiophyceae predominated in the soil algae communities. The major part of the algal biodiversity, 86.1% of all algal OTUs, could not be identified at the species level due to insufficient representation in reference sequence databases. The classes Ulvophyceae and Xanthophyceae exhibited the most unknown species diversity. About 9% of the *Meseta* algae species diversity was shared with that of the temperate reference site in Germany.

**Discussion:**

In the small portion of algal OTUs for which their distribution could be assessed, the entire ITS2 sequence identity with references shows that the soil algae likely have a wide distribution beyond the Polar regions. They probably originated from soil algae propagule banks in far southern regions, transported by aeolian transport over long distances. The dynamics and severity of environmental conditions at the soil surface, determined by high wind currents, and the soil algae’s high adaptability to harsh environmental conditions may account for the high similarity of soil algal communities between the northern and southern parts of the *Meseta*.

## Introduction

Algae and cyanobacteria are the most widespread and abundant photosynthetic life in the ice-free terrestrial ecosystems of the Antarctic (Broady and Smith, 1994; Broady, 1996; Elster and Benson, 2004). Antarctic soils represent simplified systems where microorganisms are the principal drivers of nutrient cycling. This relative simplicity makes these ecosystems particularly vulnerable to perturbations (Czechowski et al., 2016; Obbels et al., 2016; Kleinteich et al., 2017). Microalgae and cyanobacteria have been regarded as sensitive to changing environmental conditions (Davey, 1991). It makes them valuable tools for predicting the ecological consequences of global warming on Antarctic systems (Wynn-Williams, 1996a,b; Mataloni et al., 2000; Mataloni and Tell, 2002; Kleinteich et al., 2017).

In soils, the edaphic algae are concentrated in the top few centimeters of the soil profile and exposed to environmental and seasonal changes (Davey and Clarke, 1991). Those microbial communities live attached to and between soil particles, i.e., in small lacunas filled with water or with high moisture content (Bérard et al., 2005; Bonkowski et al., 2019). Recently, the global importance of soil algae in terms of abundance and global C uptake has become evident. Worldwide, soil algae take up carbon (C) in amounts equal to about 6% of the net primary production of terrestrial vegetation (Jassey et al., 2022). The soil algae and cyanobacteria provide numerous effects on the development of soils in the ice-free habitats of Antarctica. They are considered significant drivers of organic matter input into early soils (Tibbles and Harris, 1996; Tscherko et al., 2003; Frey et al., 2013; Seppey et al., 2017). Green algae, such as lichen photobionts, can contribute substantially to C production and initial soil formation (Freeman et al., 2009; Wong et al., 2010). Algae, together with cyanobacteria, influence the texture of the soils and stimulate other microbial activities. As primary colonizers of soils recently exposed to ice recession, they bind soil particles and increase aggregate stabilization (Wynn-Williams, 1990; Broady, 1996). They reduce the impact of wind erosion by forming water-stable aggregates (e.g., Metting, 1996; Büdel et al., 2016). This may promote the establishment of moss and lichen vegetation on fellfield soil surfaces in the ice-free Antarctica (Wynn-Williams, 1990; Davey and Clarke, 1991). Numerous effects on their soil habitats (Oliverio et al., 2020) are caused by the soil algae and cyanobacteria’s enormously broad biochemical diversity of pigments, photosynthetic storage products, cell walls and mucilage, fatty acids and lipids, oils, sterols and hydrocarbons, and bioactive compounds (e.g., Metting, 1981; Borowitzka, 1995). Therefore, soil algae serve as an essential food source for various small soil animals and even phagocytotic protists (Hess and Melkonian, 2013; Seppey et al., 2017). Despite the importance of soil algae for the Antarctic ice-free terrestrial systems, their diversity is only poorly known. In addition, geographic isolation, as well as human disturbance, may be key factors in understanding the biogeography of terrestrial microalgal communities in Antarctica (Chown et al., 2015).

In Maritime Antarctica, where ice has retreated, bare rock and the fine material resulting from weathering, followed by early stages of soil formation, provide a range of opportunities for algal colonization (Bölter et al., 2002). There, most soils are frost-shattered rock (barren soils) or fellfield that support only a sparse cryptogamic flora of limited taxonomic diversity and low structural complexity on its surfaces (Kennedy, 1996; Block et al., 2009; González Garraza et al., 2011; Yergeau, 2014). Alterations in the regional climate, such as the diurnal freeze-thaw cycles linked to processes of weathering (Michel et al., 2014), strong fluctuations in soil temperature (sometimes exceeding 20°C during summer and falling below –10°C during winter), and water availability, i.e., desiccation of the soils in the summer, following water saturation during spring after freezing of free water (Davey, 1991), significantly determine the biological activity and recruitment of soil microorganisms.

Previous studies on Antarctic terrestrial algae applied the traditional morphospecies concept, which requires following the algae’s development through the study in unialgal culture (e.g., González Garraza et al., 2011). However, morphological conservatism and convergent evolution toward reduced morphology make microscopic observation inappropriate for fine-scale biodiversity assessments. Identification of Antarctic terrestrial microalgae has often not been to species level [e.g., Broady, 1996; Cavacini, 2001; Adams et al., 2006; Fermani et al., 2007, and citations in Adams et al. (2006)]. That certain algal genera have not been recorded from Antarctica so far might simply be due to inadequate observations.

Our study aimed to assess the species diversity of algae in fellfield soils of Maritime Antarctica independent of cultures and as precise as possible. The objective was to test two opposing hypotheses. Maritime Antarctica’s soil algal diversity may be low due to limited sources of algae adapted to harsh environmental conditions. Those algae may originate from ice-free refugia inside Antarctica, from snowfields, or have developed from associations with lichens dominating the soil surfaces. Alternatively, the soil algal communities may develop from a continuous input of algal propagules mediated by long-distance dispersal via air atmospheric circular processes or human influence over the Southern Ocean (Smith, 1991; Vincent, 2000; Tesson et al., 2016). Also, the combination of both may promote a high algal diversity in the absence of higher plant vegetation and mycorrhiza. Therefore, we examined the surface soils of *Meseta*, an ice-free plateau mountain crest of Fildes Peninsula, King George Island, far from the seashore, representing a soil developmental gradient in a glacier forefield (Boy et al., 2016). Being openly exposed to microbial colonization from outside Antarctica and connected to the much harsher and dryer ice-free zones of the continental Antarctic (Convey, 2010) makes *Meseta* of Fildes Peninsula a promising location to test those hypotheses.

Previous studies pointed out four algal classes, i.e., the Chlorophyceae, Trebouxiophyceae, and Ulvophyceae of the Chlorophyta, and the Xanthophyceae (Stramenopiles) being dominant and most genus-rich in the top layers of fellfield soils in Antarctica (Broady, 1979, Broady, 1996; Cavacini, 2001; Fermani et al., 2007; González Garraza et al., 2011; Garrido-Benavent et al., 2020) and similar cold soil environments (Alpine glacier forefields and the Himalayas, Schmidt et al., 2010; Frey et al., 2013). Other common soil algal groups, i.e., the diatoms, Eustigmatophyceae, and the streptophyte green algae, have low diversity (e.g., Broady, 1996; Cavacini, 2001; Fermani et al., 2007; González Garraza et al., 2011) or were not detected at all (Garrido-Benavent et al., 2020) in those soil habitats. Therefore, by focusing our study on the four algal classes, Chlorophyceae, Trebouxiophyceae, Ulvophyceae, and Xanthophyceae, we anticipated capturing important key groups of the soil algal communities, except for the cyanobacteria, to be expected in the terrestrial habitats of Maritime Antarctica. With the employment of a metabarcoding analysis based on amplicons of the highly variable nuclear-encoded ITS2 rDNA region, our work may serve as a pilot study allowing for a high taxonomic resolution of Antarctic terrestrial algae with comparisons of species and even genotypes within a species. To test how much of the eukaryotic soil algae recovered from the *Meseta* of Fildes Peninsula can be found in a contrasting temperate soil environment and, therefore, obtain further insights into the distribution of *Meseta*’s soil algae distribution, we included a reference site under mild land use located in a rural region of Germany.

## Materials and methods

### Sampling sites

**FIGURE 1:**
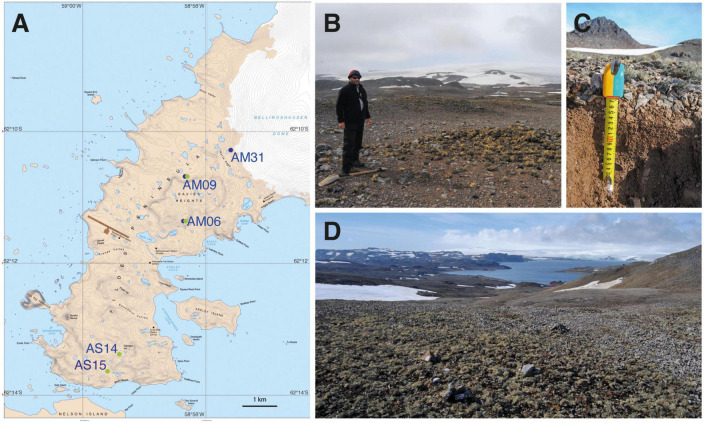
**(A)** Location of the five study sites at the southern and northern parts of the *Meseta* mountain crest of Fildes Peninsula, King George Island, Antarctica. Blue and green filled circles mark the sampling of two sampling seasons in 2013 (blue) and 2014 (green). Map No. 13799 (scale 1: 25,000) of UK Antarctic Place-names Committee, Australian Antarctic Data Centre, https://data.aad.gov.au/. **(B)** Overview of study site AM09 at Davies Heights. **(C)** Soil sampling study site AS14. **(D)** Overview of study site AS14. Note surface coverage by macroscopic lichens (*Usnea* spp.).

For assessing the genotypic diversity of soil algae from Antarctica, we selected a glacier forefield at Fildes Peninsula, located in the southwest of King George Island, Maritime Antarctica (Figure 1). Fildes Peninsula is the largest ice-free area on King George Island and was covered by glaciers until 8,000 - 5,000 years BP (Michel et al., 2014). Details about the Fildes Peninsula’s various landforms and soils as influenced by glacier retraction can be found in Michel et al. (2014) and Boy et al. (2016). The region has a cold, moist, maritime climate with a mean annual air temperature of −2.1°C (Li et al., 2014). Temperatures at Fildes Peninsula may reach 3°C maximum between December and February, with an average of 2°C in the warmest period. In the winter months (June-August) mean temperature lies at –7°C but may drop to –14°C (Braun et al., 2004). The annual precipitation at Fildes Peninsula ranges between 350 and 500 mm per year, with rainfall occurring mainly in summer (Li et al., 2014).

The soil samples used in our study originated from defined soil plots at the *Meseta*, an inland mountain crest consisting of volcanic parent rock material (Boy et al., 2016). *Meseta* receives a prolonged snow cover. The *Meseta* soil plots are located on a plateau at comparable altitudes of about 110 m a.s.l., and thus as far as possible from direct sea spray and the influence of colonies of penguins, other birds, and mammals (Boy et al., 2016; Figure 1). The latter would promote the widespread sea-to-land transfer of nutrients, enhancing vegetation growth (Michel et al., 2014; Boy et al., 2016). There was hardly any impact of bird excrement and presumably little influence by human activity. The *Meseta* may have become ice-free from the tip of the island to the glacier’s current position due to warm intervals during the deglaciation period, which started about 7,200 years ago (Watcham et al., 2011; Michel et al., 2014). The *Meseta* soil plots are along a soil development gradient formed by glacier retreat (Boy et al., 2016). It corresponds to an age-gradient of lateral direction of deglaciation as defined by radiocarbon-dated lake sediments (Watcham et al., 2011).

Our study used soil surface samples (0 – 5 cm) of five defined *Meseta* soil plots. The three sites AM31 (62.174111S, 58.923917W), AM09 (62.179694S, 58.941500W), and AM06 (62.189889S, 58.945667W) were located in the northern (younger) part of *Meseta* at North/Davis Heights in some proximity to Collins glacier and close to Profound Lake (Tiefersee) (Boy et al., 2016; Figure 1). The two sites AS14 (62.221556S, 58.978444W) and AS15 (62.225333S, 58.982778W) represented the older soils of the *Meseta* and laid close to Yanou Lake. Sediments of Yanou Lake may be dated as c. 6,200 years BP, those of Profound Lake (Tiefersee) c. 1,500 years BP (Watcham et al., 2011). All sites of the *Meseta* were openly exposed to light, seemingly providing high potential for healthy microalgae growth. Some epilithic lichen growth was observed, and lichens and bryophytes covered the soil surfaces. The plots AM06, AM09, AS14, and AS15 have been designated M2, M4, M7, and M8 in Boy et al. (2016), where further information about the surface vegetation by lichens and bryophytes and soil properties are given.

Three surface soil subsamples (0 – 5 cm) were collected at a distance of about 1 to 1.5 meters from each other within a square of about 2.5 to 2.5 meters from each of the five soil plots. Within a plot, the area of each subsample covered an area of about 10 to 10 centimeters or slightly more, yielding about 10 grams of soil. In the field, an ethanol-cleaned scalpel was stuck into the soil close to the actual sampling area and then used to take the soil surface sample. The subsamples from each plot were pooled in the field to obtain a composite sample for each plot. Two composite soil samples of the *Meseta’s* northern (AM06-14, AM09-14) and southern (AS14-14, AS15-14) parts were collected in February 2014. They were complemented by three additional composite soil samples of the northern part collected 1 year before (AM31-13, AM09-13, AM06-13). Finally, there were 21 replicate samples taken along five plots of the *Meseta* to analyze its surface soil algae community. The composite soil samples were kept frozen during the field campaign and transportation and then stored frozen at –20°C until DNA extraction.

A collective sample (consisting of five replicate subsamples) from an additional temperate soil site, *SchF*, served as a reference to identify those soil algae from a contrasting environment shared with the *Meseta.* The reference site *SchF* is in a rural region of Germany, between the housing of a small village and a small creek (Supplementary Figure 1). Without using fertilizers and pesticides, the site grew comparatively small amounts of various forage crops, which changed yearly. The site *SchF* was in the village of Schlarpe, Uslar, Germany (51.649111N, 9.750778E). The five replicate subsamples were collected in March 2015 before the vegetation started and kept frozen at –20°C until DNA extraction.

### DNA extraction, PCR, and cloning

DNA from the *Meseta* and *SchF* composite samples was extracted from the soil samples after mild cell breakage with glass beads in a Minibeadbeater cell homogenizer (Biospec, Bartlesville, OK, USA) and then using the MoBio Power Soil DNA extraction kit (MoBio Laboratories, Carlsbad, CA, USA) according to the manufacturer’s recommendations. DNA was extracted from each composite sample three times (technical replicates). The concentration of DNA extracts was quantified using a NanoDrop ND-1000 spectrophotometer (NanoDrop Technologies, Wilmington, DE, USA). We used the ITS2 rRNA gene region as a marker of high taxonomic resolution. We used clone libraries before the high-throughput sequencing to test for optimal PCR primer combinations. Initial tests with general PCR primers resulted in clone libraries comprising an algal diversity much below our expectations. Therefore, we tested the preferential amplification of targeted algal groups with various primer combinations. However, available primers recommended for targeting streptophyte green algae (Škaloud and Rindi, 2013) resulted in clone libraries, which in addition to sequences from the targeted group, also comprised those from various bryophytes as well as non-photoautotrophic protists. Bryophytes were abundant at the surface of the *Meseta* soil plots (Boy et al., 2016). PCR amplification with the following three forward primers, located at the 3′-end of the 18S rRNA gene, and combined with the general reverse primer LR1850 (Friedl, 1996), situated in the 5′-end of the 26S rRNA gene, resulted in clone libraries with almost no other sequences than those from the targeted algal groups. The forward primers were AL1500af (Helms et al., 2001), suited for the Chlorophyceae and Trebouxiophyceae (Chlorophyta), ITS-Ulva-F (Lin et al., 2012) for the Ulvophyceae (Chlorophyta), and Xits2F (Rybalka et al., 2013) for the Xanthophyceae (Stramenopiles). The group-targeted amplicons were obtained with three technical replicates from each replicate DNA extract of a composite soil sample. PCR conditions, establishment, and sequencing of the clone libraries were described previously (Rybalka et al., 2013). More than 500 clones were established and sequenced.

### Paired-end ITS2 metabarcoding and sequence processing

The group-targeted PCR revealed long amplicons (>1,200 base pairs), spanned from the 3′-end of the 18S rRNA gene over the ITS1 region, the 5.8.S rRNA gene, the ITS2 region, until the 5′-end of the 26S rRNA gene. They served as templates for a second PCR amplification, which yielded the shorter amplicons (<300 base pairs) required for the Illumina MiSeq platform. We tested various primer combinations based on clone sequences to obtain shorter amplicons, which comprised only the full ITS2 regions with adjacent portions of the 5.8S and 26S rRNA genes. The general forward primer 5.8SbF (Mikhailyuk et al., 2008) combined with the reverse primers ITS4 (White et al., 1990) for the green algal (Chlorophyta) and ITS4Xan (Rybalka et al., 2013) for the Xanthophyceae amplicons were most successful. The PCR conditions for the amplification of the short amplicons were as follows: initial denaturation at 95°C for 5 min, followed by 20 cycles of 95°C for 30 s, 51°C for 30 s, and 72°C for 1 min, and final elongation at 72°C for 5 min. Three technical replicates for each of the short amplicons were performed. The short green algal and Xanthophyceae amplicons were equimolarly pooled after quantification with the Qubit dsDNA HS Assay Kit (Life Technologies, Carlsbad, CA, USA) and subjected to library preparation as previously described (Meyer and Kircher, 2010; Kircher et al., 2012). Sequencing was performed on an Illumina MiSeq platform (2 × 250 bp) at the Transcriptome Analysis Laboratory (*TAL*) at the University Medical Center Göttingen, Department of Developmental Biochemistry, University of Göttingen (Germany).

The raw sequence data were received demultiplexed, i.e., already split into separate files for each sample. A total of 1,347,028 raw reads were obtained from the seven *Meseta* samples and 197,335 raw reads from the *SchF* sample. Sequences with wrong or incomplete indexes or primers were discarded using our own Perl script. The script also separated the sequence runs from the Xanthophyceae amplicons from those of the green algal amplicons. After removing the primers and adapters, the sequences were re-orientated into a 5′-3′ direction where needed. The pair-end reads were joined using PEAR (Zhang et al., 2014), and the assembled sequences were then filtered with VSEARCH (Rognes et al., 2016) to improve the data quality. All sequences that were longer than 200 bp and with the maximum expected number of errors smaller than 1 were retained in VSEARCH as recommended by Edgar and Flyvbjerg (2015). Next, VSEARCH was used to dereplicate the identical sequences and for the subsequent *de novo* chimera detection. Finally, 579,046 and 94,971 processed reads from the *Meseta* and *SchF* samples were available for downstream analyses. The ITS2 regions were extracted with ITSx version 1.1b (Bengtsson-Palme et al., 2013; Rivers et al., 2018), multiplexed again, and clustered into Operational Taxonomic Units (OTUs). The latter was performed with VSEARCH using an identity threshold of 0.97 and preliminary sorting by decreasing input sequence abundance. The cluster’s determination with the most similar centroid sequence for each sequence was obtained with the options *maxaccepts* and *maxrejects* set to 0. We excluded those OTUs with representative sequences less than 0.005% of the initial read numbers (Bokulich et al., 2012). Finally, BLASTN Version 2.10.1 + with standard settings (Altschul et al., 1997) was used to query each OTU representative against the whole GenBank Nucleotide database (NCBI-GenBank Flat File Release 252.0 of October 15, 2022) and the first 50 hits were recorded into a reference table (*blastout* table). We determined a distinct taxonomic label for each OTU using a consensus approach by assigning the best supported taxonomic rank across the first 10 BLAST hits with respect to their bit scores, which BLASTN calculates in database queries (Altschul et al., 1990; see Supplementary File 1 for a detailed description). In addition, a manual examination and analysis of all recorded BLAST hits were performed in case of ambiguity. To facilitate this comparison between OTUs and putative reference sequences, we normalized each bit score S’ with regard to the corresponding reference sequence length. The normalized bit score (referred to as *NB*, *N*ormalized *B*it score, in the following) reflects a combination of the fields “sequence identity” and “query cover” of BLAST queries and allows to compare the query-reference similarities for reference sequences of differing lengths. Furthermore, it enables the convenient graphical display of the similarities using box plot diagrams. The *NB* was maximal (*NB* = 1.81) using BLASTN at full identity of the paired sequences, i.e., 100% query cover, 100% sequence identity, and zero E value.

### Statistical analyses

All statistical analyses were performed using R [Version 4.0.2; R Core Team (2020)] and utilizing the packages *tidyverse* (Version 1.3.1; Wickham et al., 2019), *phyloseq* (Version 1.36.0; McMurdie and Holmes, 2013), *RColorBrewer* (Neuwirth, 2022), and *vegan* (Version 2.5-6; Oksanen et al., 2019). Rarefaction curves were calculated and visualized with *vegan*. Relative abundances, alpha diversity indices, and heatmaps were computed with *phyloseq*. The Venn diagram was calculated with *VennDiagram* (Chen and Boutros, 2011) and *ggVennDiagram* (Gao et al., 2021) of *tidyverse*. Graphical display of the rarefaction curves, the box plot diagrams of alpha diversity indices, and the *NB* values of the algal OTUs were done with *ggplot2* (Wickham, 2016) of *tidyverse*. To assess the differences in alpha diversity between sites, non-parametric Kruskal–Wallis tests were performed. All scripts and the Perl script used to separate Xanthophyceae sequence runs from those of green algae (including other functions) are available on the following GitHub repository: https://github.com/daniel-nimptsch/antarctic_project_tf.

## Results

### Diversity of targeted classes of eukaryotic algae

**FIGURE 2:**
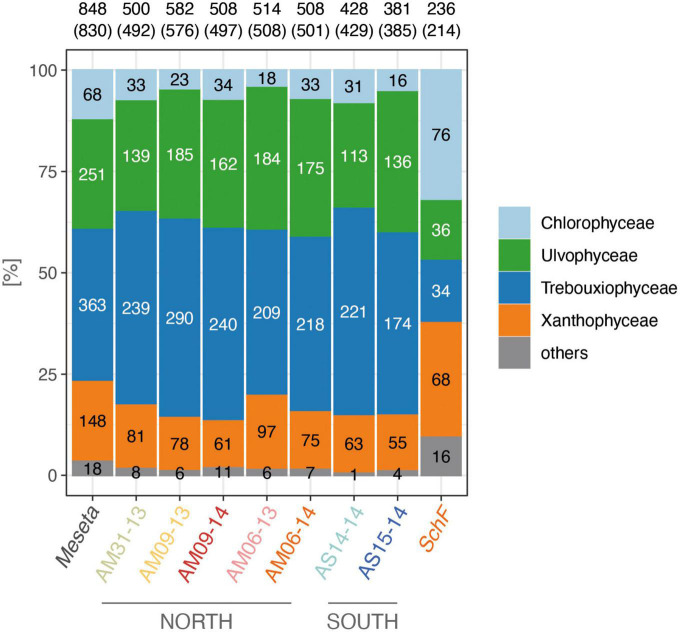
Relative OTU counts of studied samples from the northern and southern parts of *Meseta* of Fildes Peninsula, King George Island, Antarctica, and the temperate reference site *SchF* (Uslar, Germany). Most-left column, arithmetic means of the *Meseta* samples. Numbers are the absolute numbers of OTUs, those in brackets only of the algal OTUs.

The paired-end sequencing approach revealed 848 OTUs for the seven *Meseta* samples (Supplementary Table 1). Their sequences comprised ITS2 regions of variable lengths, i.e., 174–295 bp (average 228 bp). 830 OTUs (97.9%) represented the four targeted algal classes. Trebouxiophyceae was, with 363 OTUs (43.7% of all algal OTUs), the most diverse algal class at the *Meseta*, and the Chlorophyceae, with just 68 OTUs (8.2% of all algal OTUs), the least diverse class (Figure 2). Only 18 OTUs (2.1% of all algal OTUs) represented organisms other than algae (bryophytes, fungi) or were left unassigned. The sample from the temperate reference site *SchF* (Uslar, Germany) had only 214 algal OTUs, which is about one-quarter (25.8%) of all *Meseta* algal OTUs and half (55.6%) of that of the *Meseta* sample with the lowest number of algal OTUs, AS15-14 (Figure 2). In contrast to *Meseta*, at site *SchF*, the Chlorophyceae was the dominant algal group (35.5% of all *SchF* algal OTUs), while Trebouxiophyceae and Ulvophyceae had the lowest numbers of OTUs. The relative abundance of Xanthophyceae increased much compared to *Meseta*, i.e., from 17.8% at the *Meseta* to 31.8% at *SchF* (Figure 2).

**FIGURE 3:**
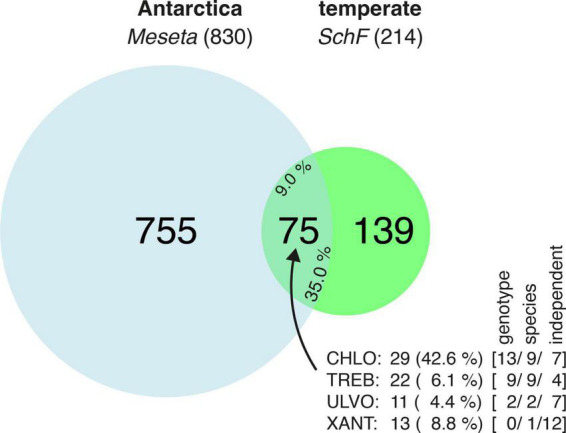
Venn diagram which shows the proportion of algal OTUs recovered from the *Meseta* of Fildes Peninsula, King George Island, Antarctica, that of the temperate references site *SchF* (Uslar, Germany), and the proportion of the OTUs shared by both localities (overlap). The total number of shared OTUs is also expressed in percentages for the *Meseta* **(left)** and *SchF* OTUs **(right)**. The insert depicts the composition (in total numbers and percentages) of the 75 OTUs in the overlap with respect to the four targeted algal classes (CHLO, Chlorophyceae; TREB, Trebouxiophyceae; ULVO, Ulvophyceae; XANT, Xanthophyceae). In square brackets, numbers of OTUs identified at the level of genotypes (entirely identical with a reference sequence, *NB* = 1.81), species (identity with a reference sequence at 1.75 ≤ *NB* < 1.81), and of those OTUs independent of reference sequences.

Sequence reads from *Meseta* and *SchF* were simultaneously processed and finally clustered into OTUs (97% similarity threshold). There was an overlap of 75 OTUs shared between the *Meseta* and the reference site *SchF* (Figure 3). It equals about one-third (35.0%) of the recovered *SchF* algal OTUs and almost one-tenth (9.0%) of the recovered algal OTUs from the *Meseta* soil plots. Interestingly, a large fraction, 42.6% (29) of all (68) *Meseta* Chlorophyceae OTUs were within the overlap. In contrast, the other classes’ overlap OTUs formed much smaller fractions of ≤8.8% (Figure 3). It may be explained by Chlorophyceae being the largest algal group at the reference site *SchF* in contrast to the *Meseta* (Figure 2). About one-third of all overlap OTUs (32.0% or 24 OTUs) had entirely identical ITS2 sequences with available references (*NB* = 1.81). All those OTUs represented algal genotypes identical to those already recovered from geographical regions other than the Polar regions (Table 1 and Supplementary Table 2). They included, for example, genotypes of *Coccomyxa subellipsoidea, Pseudostichococcus monallantoides, Raphidonema sempervirens*, some lichen photobionts of the genera *Asterochloris*, *Trebouxia*, and *Tetradesmus obliquus* which based on available reference sequences have already been recovered >10 times in various geographic regions. Of all the overlapping OTUs, 30 (40.0%) could not be identified due to the unavailability of close references (Supplementary Table 3). Nonetheless, their distribution was deduced as sequence reads from both the *Meseta* and *SchF* were grouped into the same OTU. Those unidentified OTUs made up 14% of all *SchF* algal OTUs. Finally, 21 (28.0%) of all overlapping OTUs were not identical to available sequences but highly similar, i.e., 1.75 ≤ *NB* < 1.81, so they might be regarded as the same species (Table 1). Notably, 36 (48.0%) of all overlapping OTUs were found in both parts of the *Meseta* (Supplementary Table 4). These findings suggest a connection between the algae communities in the *Meseta* and those in temperate areas.

**TABLE 1:** Taxonomy of the 115 algal OTUs retrieved from the *Meseta* of Fildes Peninsula identified to species, with full (genotype) or high (species level) identity with references, and their distribution.

Class	Identity level	*Meseta* only	Overlap *Meseta*/*SchF*	Polar only
Chlorophyceae (39)	genotypes^1^ (18)	*Coelastropsis costata, Tetradesmus obliquus*	*Chlorosarcinopsis eremi*, *Coelastrella striolata*, *Coleochlamys apoda*, *Desmodesmus denticulatus*, *Heterochlamydomonas* sp., *Hormotilopsis gelatinosa*, *Spongiococcum aplanosporum*, *Tetracystis sarcinalis*, *T. vinatzeri*, unident. Chlamydomona-dales, unident. chlorophyte (3)	*Chlorominima collina, Chodatodesmus australis, Coenochloris* sp.
	species^2^ (21)	*Bracteacoccus aggregatus, B. bullatus* (2), *Bracteacoccus* sp., *C. oocystiformis*, *Coelastrella striolata*, *Coenochloris* sp., *Sanguina aurantia, S. nivaloides*, unident. Chlamydomonadales (2), unident. chlorophyte	*Chlorococcum* sp. (3), *Coelastrella aeroterrestrica, Coelastrella* sp., *Spongiochloris spongiosa, Spongiochloris* sp., unident. Chlamydomonadales (2)	n.a.
Trebouxiophyceae (65)	genotypes^1^ (28)	Asterochloris pseudoirregularis*, A. stereocaulonicola*, Chloroidium lichenum*, *Diplosphaera* sp., *Edaphochloris andreyevii, Elliptochloris* sp.*, Myrmecia pyriformis*, *Raphidonema catena*, *R. sempervirens*, Trebouxia suecica*, T. vagua*, unident. Chlorellales, unident. trebouxiophyte (3)	*Apatococcus* sp., *Chlorella vulgaris* (2), Coccomyxa subellipsoidea*, *Elliptochloris* sp.*, *Laetitia sardoa*, *Parietochloris bilobata, Pseudostichococcus monallantoides* (2)	Chloroidium antarcticum*, *Chloroidium* sp., *Raphidonema nivale*, Stichococcus antarcticus*
	species^2^ (37)	Coccomyxa subellipsoidea*, Coccomyxa sp. (2), *Deuterostichococcus allas, Diplosphaera chodatii* (2)*, Elliptochloris subsphaerica, *Elliptochloris* sp., *Lobosphaera* sp., *Muriella terrestris, Myrmecia* sp.*, *Neocystis mucosa*, *Neocystis* sp., *Pseudochlorella signiensis, Pseudochlorella* sp., *Stichococcus* sp. (2) Trebouxia impressa*, Trebouxia sp. (3)*, unident. Chlorellales, unident. trebouxiophyte (5)	*Coccomyxa viridis, Elliptochloris subsphaerica, Elliptochloris* sp., *Muriella terrestris, Myrmecia* sp. (2)*, *Neocystis mucosa*, unident. Chlorellales, unident. trebouxiophyte	Coccomyxa antarctica*
Ulvophyceae (8)	genotypes^1^ (3)	n.a.	*Chamaetrichon basiliense*, *Planophila laetevirens*	*Protomonostroma dakshina*
	species^2^ (5)	*Planophila bipyrenoidosa*, *Planophila* sp., *Urospora* sp.	*Chamaetrichon* sp., *Planophila laetevirens*	n.a.
Xanthophyceae (3)	genotypes^1^ (0)	n.a.	n.a.	n.a.
	species^2^ (3)	*Heterococcus conicus*, *H. viridis*	*Heterococccus virginis*	n.a

^1^, entire sequence identity (*NB* = 1.81) with reference. ^2^, species identity level (1.75 ≤ *NB* < 1.81). asterisk, lichen photobiont.

The clone library approach revealed long sequences that spanned from the 3′-end of 18S over the ITS1, the 5.8S, and the ITS2 regions to the 5′-end of the 26S rRNA gene. A total of 235 cloned ITS2 algal sequences clustered together with the paired-end sequence reads into 113 OTUs. Those were mixed OTUs, i.e., comprising sequences of both approaches (Supplementary Table 1). There were 93 mixed algal OTUs (11.2% of all OTUs) from the *Meseta.* About half (46) of those included more than one clone sequence, i.e., up to 14 clone sequences per OTU (Supplementary Table 1). A total of 15 mixed OTUs were from the overlap between *Meseta* and *SchF.*

### Taxonomic composition of the fellfield soil algal communities

**FIGURE 4:**
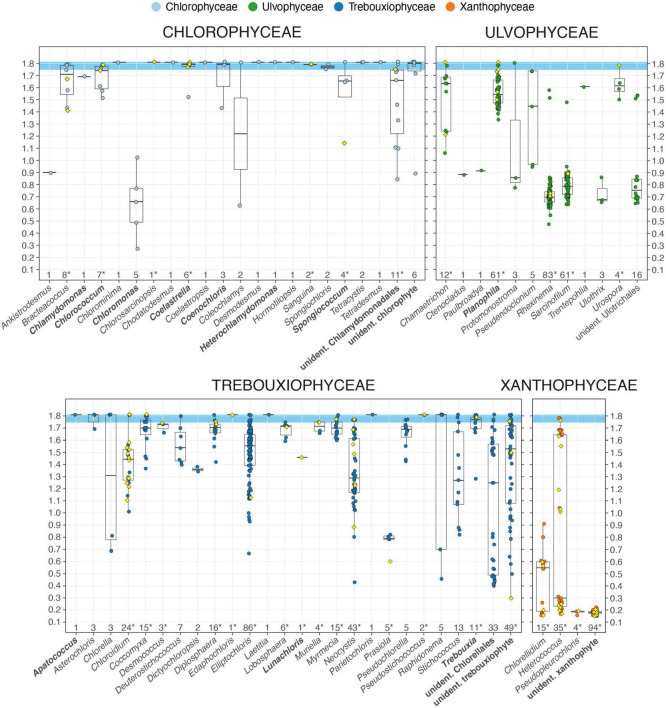
Boxplot graphical display of the 830 algal OTUs recovered from the *Meseta* of Fildes Peninsula. Each colored marker represents an OTU and a diamond-shaped marker, an OTU that includes sequences from the clone library approach (mixed OTU). The OTUs are arranged into sets that represent 58 algal genera and six groups of unidentified OTUs distributed among the four targeted algal classes. The OTU’s value of the pairing significance with its closest reference (normalized bitscore, *NB*), range 0.1–1.81, defines its position along the *x*-axis. The boxplot displays the OTUs within the range of the *NB* values of a set of OTUs. For an OTU set the boxplot’s horizontal lines represent the median of all *NB* values, the first and the third quartiles. A whisker extends from the smallest and the largest value to the first or third quartile if the values are within 1.5 times the distance to the quartile (inter-quartile range, IQR). Values beyond that point (outliers) are plotted individually, or no whisker is shown. Numbers of OTUs per set are given on the y-axis, and an asterisk marks those sets (genera) with mixed OTUs, i.e., those which include sequences from the clone library approach. Names in bold mark OTU sets which include OTUs shared between the *Meseta* and the temperate reference site *SchF*.

The box plot diagram of Figure 4 displays the distribution of the 830 *Meseta* algal OTUs with their corresponding *NB* pairing scores across 58 genera of the four targeted algal classes. Assignment of an OTU to a genus was based on a consensus approach to determine a distinct taxonomic label for each OTU (see Supplementary File 1). In those cases where an OTU had multiple hits with differing taxonomic labels for the genus, the genus with the highest sum of bit scores of all hits referring to that genus was assigned. Within each genus with *n* ≥ 2 OTUs, *NB* pairing scores of the OTUs varied substantially: For example, the *NB* values of the 86 OTUs of *Elliptochloris* (Trebouxiophyceae) ranged from 1.81 (full sequence identity) down to 0.64 (Figure 4 and Supplementary Table 1). For each OTU within that range, most of the top 10 or 50 recorded BLASTN hits referred to *Elliptochloris*, and thus, that genus received the highest sum of bit scores and was also assigned by the consensus method. In each of the Chlorophyceae, Trebouxiophyceae, and Ulvophyceae, there were groups of OTUs that could be identified only to the level of orders, i.e., the Chlamydomonales, the Chlorellales, and the Ulotrichales by the sequence comparisons. Four more sets of OTUs were assigned only classes due to the lack of reference sequences. Those were the unidentified chlorophyte, the unidentified ulvophyte, the unidentified trebouxiophyte, and the unidentified xanthophyte (Figure 4). The set of unidentified xanthophyte OTUs was assigned to class only due to the fact that their amplification with the PCR primers was primarily effective for targeting the Xanthophyceae.

The temperate exemplar site *SchF* shared 14 of the 58 genera with the *Meseta* (Figure 4). At site *SchF* there were only three unique genera, i.e., the chlorophytes *Desmotetra, Fasciculochloris, Neochlorosarcina*, and the ulvophyte *Tupiella*, all of which were not found at the *Meseta* (Supplementary Table 1). The 93 mixed *Meseta* OTUs (which included sequences from the clone library approach) were distributed on 31 of the 58 algal genera. The clone libraries recovered some additional genera from the *Meseta* not found by the paired-end sequencing. Those were the chlorophyte *Graesiella emersonii* (*NB* = 1.79), the trebouxiophyte *Watanabea* sp. (*NB* = 1.75), the xanthophyte *Pleurochloris* sp. (*n* = 17; *NB* = 1.60 - 1.71), unidentified xanthophytes (*n* = 18; *NB* = 0.27 - 0.70), and the streptophyte green algae *Cylindrocystis* sp. (*NB* = 1.25), *Interfilum massjukiae* (*n* = 2; *NB* = 1.79), *Interfilum* sp. (*n* = 4; *NB* = 0.64 - 1.74), and *Klebsormidium* sp. (*n* = 20, *NB* = 1.71 - 1.81). The paired-end sequencing may have omitted them due to having ITS sequences that were too long. Furthermore, they were amplified by PCR primers only used in testing for the optimal primer combination, but due to their low performance, were not employed in the paired-end approach.

We considered an OTU to represent the same species as its closest reference when their sequence alignment was at *NB* ≥ 1.75 (Figure 4). Those high identity values usually corresponded to high query coverages of >95% and sequence identities of ≥96%, corresponding to about a four to six positions difference between two sequences (average length of 228 base pairs). Such small sequence divergences may well be within the sequence variation of a species with respect to the rapidly evolving ITS2 marker. Finally, we regarded the entire sequence identity with a reference sequence (*NB* = 1.81) to represent the same genotype and top matches within the range of 1.75 ≤ *NB* < 1.81, the same species. In the Chlorophyceae and Trebouxiophyceae, almost every genus had OTUs with genotype identity and/or within the species range. In contrast, the Ulvophyceae had only four genera (eight OTUs), and the Xanthophyceae had only one genus (three OTUs) with OTUs within that range (Figure 4). It demonstrates an important lack of appropriate close references in these algal classes. In the Xanthophyceae, confirmation of class assignment was provided by mixed OTUs independent of the consensus approach. There, 20.3% of all OTUs were recovered through cloning. Then aligning of the 3′-end of the 18S rRNA gene sequence with references was utilized to ensure the correct class assignment. It was critical where the consensus approach failed due to the lack of significant alignments with references.

For the groups of unidentified OTUs, identification was impaired despite high similarities to reference sequences from databases. Either the closest reference sequence has not been identified to genus but only to class (e.g., “unidentified trebouxiophyte”), order (e.g., “unidentified Chlamydomonadales,” and “unidentified Chlorellales”) (Figure 4). Several top matches (*NB* ≥ 1.75) were with those closest reference sequences not referring to a certain species (Table 1 and Supplementary Table 1). Those were mostly from uncultured environmental material. For example, the OTU_0641 was assigned *Elliptochloris* sp., although it had maximum identity with a reference referred to as “uncultured Chlorophyta clone” (sequence acc. no. MH258956). Out of the 50 BLASTN hits of that OTU, the entries referring to *E. subsphaerica* received the highest sum of bit scores. However, there was *NB* = 1.73 with the closest reference referring to that species. Therefore OTU_0641 and sequence MH258956 were regarded not to represent that same, *E. subsphaerica*, but rather a different yet-to-be-identified species of *Elliptochloris*. The databases often did not recognize the reference sequences from uncultured environmental material as originating from algae. Instead, they were mainly erroneously assigned to fungi. However, their algal origin became evident when the subsequent more distant references were from algae, as revealed upon manual inspection of the best 50 BLAST hits (*blastout* table). For example, for OTU_0006, of the 50 recorded BLASTN hits at sequence coverages ≥95%, 21 entries were referred to uncultured fungi (likely misidentified as such) and 29 to green algae, mostly Ulvophyceae. The sum of the bit scores for the Ulvophyceae entries was higher than those for the uncultured fungi. Within the Ulvophyceae group, those entries referring to the genus *Planophila* (*n* = 10) had a higher bit score sum compared to the other entries. Still, sequence identities with any species in that genus were with *NB* < 1.5. As a result, OTU_0006 was assigned *Planophila* sp. (Ulvophyceae).

Entire ITS2 sequence identity with available references (*NB* = 1.81) was found only for a small fraction of 5.9% or 49 of all *Meseta* algal OTUs (Figure 4, Table 1, and Supplementary Table 2). In those cases, there was genotypic evidence for the distribution of most *Meseta* soil algae. Most of their references have been obtained from cultures or environmental material originating from outside Antarctica (Supplementary Table 2). For example, the OTU_0005 recovered from all *Meseta* sites shared full ITS2 identity with the green algal culture strain CCAP 250/1 *Myrmecia pyriformis* isolated from Austria (sequence acc. no. MW471028; Supplementary Table 2). It also shared full identity with two unidentified clones (sequence acc. no. FJ554300 and MG207147) from environmental studies on forest sites in Canada (Hartmann et al., 2009) and the USA (Bullington, 2017). We regarded a little lower similarity with references, i.e., 1.75 ≤ *NB* < 1.81, still within the ITS2 sequence variation of a species (Figure 4 and Table 1). Another small fraction, i.e., 8.0% or 66 of all *Meseta* algal OTUs, were within that range and could be assigned to species. We conclude that the sequence comparisons provided an unambiguous taxonomic assignment, i.e., at genotypic or species identity, only to a fraction of 13.9% or 115 of all *Meseta* algal OTUs (Figure 4, Table 1, and Supplementary Table 1).

### Algal community composition along the Fildes peninsula mountain crest

**FIGURE 5:**
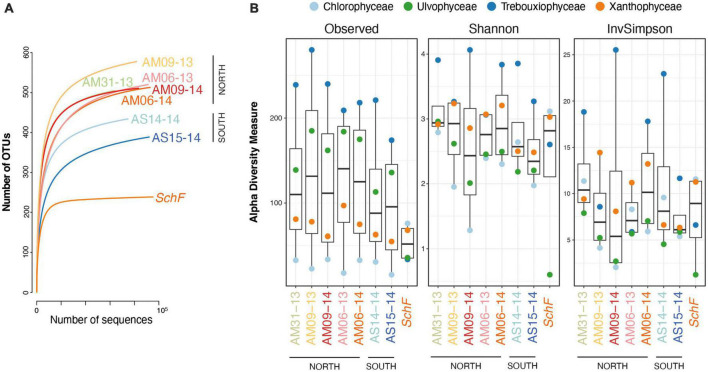
Diversities of the seven samples from the *Meseta* mountain crest of Fildes Peninsula, Antarctica, and the temperate reference site *SchF*. The two parts of the *Meseta* are indicated (see Figure 1A). **(A)** Rarefaction curves of the total OTU numbers. All samples have been sequenced to near-asymptote. **(B)** The alpha diversity indices Observed, Shannon and InvSimpson of the seven *Meseta* samples and reference site *SchF are* shown in boxplots.

The five studied sites of the *Meseta* were highly similar in their diversity of soil algal communities. The relative OTU counts varied slightly among the seven *Meseta* soil samples (Figure 2). The samples from the two southern sites of *Meseta*, AS14 and AS15, had lower total OTU numbers (mean 407) than the three sites in the northern part, AM31, AM09, and AM06 (mean 522; Figure 2). The rarefaction curves for richness indicated lower diversities for the southern than the northern plots. The exemplary temperate site *SchF* had the lowest richness (Figure 5A). The alpha diversity of the four targeted algal classes was calculated using the indices Observed, Shannon, and InvSimpson and is depicted in a boxplot (Figure 5B). No significant differences existed among the seven samples from *Meseta* and the sample from reference site *SchF* (*p* < *0.05*; non-parametric Kruskal–Wallis test).

**FIGURE 6:**
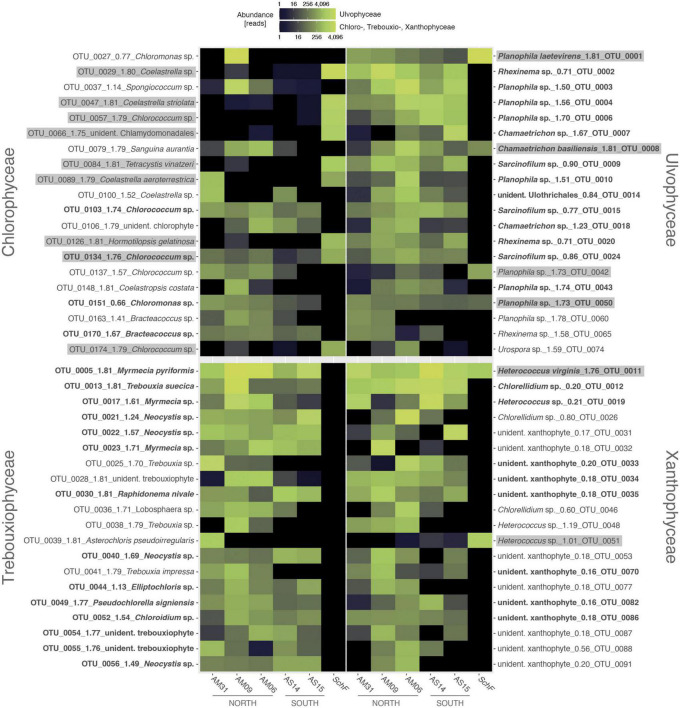
Heat maps of abundances of the top 20 algal OTUs per class show their distribution along the five sites of *Meseta* of Fildes Peninsula, King George Island, Antarctica, and the temperate reference site *SchF.* An OTU ID is with its species identification and the normalized score of pairing significance to its closest reference sequences (*NB*; see Figure 4). In bold are OTUs recovered throughout all five *Meseta* sites. Highlighted in gray are OTUs recovered from all five *Meseta* sites, as well as site *SchF*. Scale, color brightness within the matrix indicates the absolute sequence reads.

All algal OTUs per class were checked with respect to their distributional pattern at both parts of the *Meseta* and the temperate reference site *SchF*. Heatmaps visualized the distribution of the 20 OTUs with the highest read numbers (top 20 OTUs) from each algal class (Figure 6). There were no discernible distributional patterns of OTUs observed at the five Meseta sites. For the Chlorophyceae, the heatmap reflected that the class encompasses the highest percentage (42.6%) of OTUs shared between *Meseta* and the temperate reference site *SchF* (overlap OTUs; Figure 3 and Supplementary Table 4). In contrast, the fraction of the overlap OTUs was ≤8.8% in the other three classes. The top 20 OTUs of Chlorophyceae included nine such shared OTUs, while there were four, two, or none of those OTUs in the Ulvophyceae, Xanthophyceae, and Trebouxiophyceae (Figure 6). Interestingly, many overlap OTUs had a higher abundance (number of reads) at the site *SchF* than at the *Meseta*, e.g., OTU_0001 *Planophila laetevirens*, OTU_0051 *Heterococcus* sp. and OTU_0047 *Coelastrella striolata* (Figure 6). A total of 36 OTUs from all four targeted algal classes were found in both the northern and the southern part of the *Meseta*, as well as the temperate reference site *SchF* (Supplementary Table 5). Two OTUs were remarkable as they were retrieved in high abundances across all five *Meseta* sites and the temperate site *SchF*, i.e., OTU_0011 *Heterococcus virginis* (a species so far known only from Antarctica) and OTU_0001 *Planophila laetevirens* (Figure 6).

## Discussion

### Unrecognized soil algal diversity at *Meseta*

Our study revealed a considerable yet unidentifiable component of soil algae biodiversity in the topsoils of the *Meseta* mountain crest on Fildes Island, Maritime Antarctica, that remains to be characterized, i.e., for which closest references still have to be established. There were no close representatives within common databases for the vast majority of the recovered algal OTUs (NCBI GenBank). However, the taxonomic coverage varied considerably among the four targeted algal classes. Chlorophyceae may be the taxonomically best-studied class of soil algae, i.e., the *Meseta* Chlorophyceae best fitted the references from the sequence databases. In contrast, only sparse fits to the available reference sequences were for the Ulvophyceae and Xanthophyceae, demonstrating that they exhibit the most extensive still-unknown species diversity yet to be studied. Inadequacy of taxonomic coverage is a significant obstacle in the metabarcoding approach impairing its appropriate use for the species rank (Machado-de-Lima et al., 2019; Salmaso et al., 2022). Metabarcoding will remain one of the principal methods for community analyses, although a future shift toward PCR-free metagenomics and transcriptomic approaches can be expected (Salmaso et al., 2022). PCR-free metagenomics has already been employed on Polar soil algae (Rippin et al., 2018). More culturing efforts are needed to increase the coverage of reference taxonomic databases for a more efficient taxonomy annotation (Salmaso et al., 2022). Although the morphological approach based on cultures conceals significant biological and phylogenetic diversity (Machado-de-Lima et al., 2019), only cultures provide defined material for sequencing multiple markers or genomes and the characterization of taxonomic traits of the soil algal species. Most algae culture isolates from Antarctic soils in available public culture collections (e.g., the SAG culture collection) are mesophilic and can be maintained at ambient temperatures. However, there may still be a hidden diversity of cold-adapted and likely cryophilic specialist photoautotrophic microbial life in Antarctic soils and similar barren soils, which has yet to be cultured and described taxonomically (Frey et al., 2016). A considerable portion of the recovered *Meseta* soil algal OTUs without close references could still be unrecognized species with specific traits toward the adaptation to cold habitats.

Green algae (Chlorophyta), particularly the class Ulvophyceae, and the Xanthophyceae (Stramenopiles), have previously been reported as probably being the most dominant eukaryotic algal groups of Antarctic and similar barren soils (e.g., Freeman et al., 2009; Schmidt et al., 2010; Frey et al., 2013; Novis et al., 2015). Those studies considered the Ulvophyceae common in extremely cold terrestrial habitats but one of the least known algal groups from terrestrial habitats (Schmidt et al., 2010). However, using a culture-based approach only, members of Ulvophyceae likely have often been overlooked or misidentified as members of Chlorophyceae (Škaloud et al., 2013; Darienko and Pröschold, 2017). For the Ulvophyceae and Xanthophyceae, we used special group-targeted PCR primers to increase the amplification of both. Those amplicons revealed an astonishing diversity of OTUs of both classes. A higher diversity at the species level may be recovered when combining PCR amplicon metabarcoding with specific lineage-targeted primers (e.g., Fawley et al., 2021). The specific primer combination for Xanthophyceae was particularly successful as almost no other sequences (e.g., fungi or other eukaryotes) have been amplified. Employing suitable group-targeted PCR primers may be crucial to recovering the Xanthophyceae biodiversity in soil samples adequately. Recent NGS metabarcoding studies have failed to recover the Xanthophyceae in Antarctic terrestrial environments using general (no-group-targeted) PCR primers (Czechowski et al., 2016; Câmara et al., 2020; Garrido-Benavent et al., 2020). However, using PCR-free metagenomics, Xanthophyceae and Ulvophyceae were found to be dominant in soil crusts in the Polar regions (Rippin et al., 2018). Underestimating biodiversity due to low taxonomic resolution and insufficiently conserved primer binding sites across broad taxonomic groups may be the main pitfall in applying universal PCR primer pairs in NGS metabarcoding of Antarctic soil environments (Czechowski et al., 2017). Xanthophyceae are pioneers in colonizing early soils and out-competing other algae (Rybalka et al., 2020, 2022). Indeed, the Xanthophyceae have significant yet unrecognized roles in colonizing Antarctic ice-free soils. However, due to the current sparse availability of references, the Xanthophyceae diversity and its significance in soil processes are likely underestimated.

A low diversity of Trebouxiophyceae comprising just lichen photobionts has previously been reported from the cold-soil environments of Alpine glacier forefields (Frey et al., 2013, 2016) and the dry valleys in the high Himalayas, Arctic, and Antarctica (Fell et al., 2006; Schmidt et al., 2010). Our study also recovered several lichen photobionts, e.g., those of the genera *Asterochloris, Chloroidium, Coccomyxa, Diplosphaera, Elliptochloris*, and *Trebouxia* (Table 1). Lichens are the main component of the macroscopic vegetation on the *Meseta* soil surfaces (Boy et al., 2016). Small lichen fragments or their symbiotic reproductive propagules may have drifted into the soil by wind blow or melted snow. The lichens’ dry symbiotic diaspores provide the photobiont dispersal, inside which a fungal mycelium protects the alga. Due to their low weight, they are well suited for dispersal by wind, but birds may also be involved. Trebouxiophyceae and Chlorophyceae were jointly amplified using the same PCR primer combination in our metabarcoding approach. The Trebouxiophyceae was, in terms of OTU richness, the predominant and most diverse targeted class of soil algae, with proportions about 6–10 times higher than the Chlorophyceae at the *Meseta* study sites. Trebouxiophyceae even outcompeted Ulvophyceae and Xanthophyceae, albeit the employment of group-targeted PCR has enhanced the diversity of the latter two classes. In contrast, at the temperate exemplar site *SchF*, the proportion of Trebouxiophyceae was only about half that of the Chlorophyceae. The predominance of Trebouxiophyceae may be a characteristic feature of photoautotrophic life in the first few centimeters of Maritime Antarctica’s soils. We anticipate additional studies at other ice-free sites of Antarctica and temperate regions to substantiate that view further. The high diversity of Trebouxiophyceae at the *Meseta* sites may not only be due to preferably symbiotic species from lichen symbioses. Rather the success of the class may be because it features a broad range of species with optimal adaptation to the harsh environmental conditions of Antarctic soils, such as freeze tolerance coupled with resistance to desiccation and extended periods of darkness.

Another source of algae for the ice-free soils of Maritime Antarctica may be snow fields. The Chlorophyceae are well known as the prevalent group of snow algae (e.g., Segawa et al., 2018). They have been found to dominate the algal blooms of colored snow in Fildes Peninsula (Soto et al., 2020, 2022). At the *Meseta* of Fildes Peninsula, however, we recovered only a few Chlorophyceae genera associated with snow habitats, such as *Chlorominima* (Gálvez et al., 2021), *Raphidonema* spp., and unidentified Chlamydomonadales. All the other Chlorophyceae were genera of typical soil algae, such as *Bracteacoccus*, *Desmodesmus, Coelastrella*, and *Tetracystis* (Table 1).

### Distribution of the *Meseta* soil algae

The surprisingly high algal diversity at the *Meseta* may be because it is open to colonization from other continents, i.e., temperate regions, and connected to the much harsher and dryer ice-free zones of Continental Antarctica. At the Fildes Peninsula, climatic conditions prevail that are not as harsh as in the more remote regions of Continental Antarctica. Therefore, it offers transportation-resistant soil algae of worldwide distribution to proliferate during Austral summer. However, Fildes Peninsula also provides permafrost environments (Michel et al., 2014) to which only specialized algae may be adapted. For a small portion of *Meseta* algal OTUs, i.e., 115 or 13.9% of all 830 OTUs, our study revealed the *Meseta* soil algal community is composed of a mixture of algae that can also be found in temperate regions and few specialists that might be indigenous (Figure 3, Table 1, and Supplementary Tables 2, 4). However, the number of identifiable species may increase with an increasing number of studies on soil algae; it will also enlarge our knowledge of the distribution of Antarctic soil algae. We noted that with new releases of the NCBI-GenBank database, the number of the *Meseta* OTUs identified to species increased from 95 in 2021 (release 244.0) to 115 in 2022 (release 252.0). A fraction of 41 OTUs had entire sequence identity (*NB* = 1.81), i.e., shared the same ITS2 genotypes with references recorded from geographic regions other than the Polar regions. Several of those *Meseta* algal genotypes (OTUs) were found in non-Polar regions more than 50 times, e.g., *Trebouxia suecica, Tetradesmus obliquus*, and *Chlorella vulgaris* (Supplementary Table 2). This suggests those algae may have colonized the *Meseta* from external sources. They may be widely distributed, also in different contrasting environments. The same *C. vulgaris* genotypes found in *Meseta*, our study also recovered from the temperate reference site *SchF.* One was identical to that of a culture strain from forest soils in Germany (Hodač et al., 2016). Of the 41 OTUs with entire (genotypic) sequence identity to references outside the Polar regions, 24 OTUs were from the overlap between the *Meseta* and the reference site *SchF* (Supplementary Table 2). In the following, we give five examples of those overlap OTUs. There was an entire ITS2 sequence identity of OTU_0640 with an uncultured clone representing a species of *Apatococcus* from air-exposed green biofilms covering artificial hard substrates (Hallmann et al., 2016) or treebark (sequence accession no. ON119418) in Germany. The same genotype has already been retrieved from an airborne snow sample making its long-distance aeolian dispersal very likely (Tesson and Šantl-Temkiv, 2018). Similarly, the same genotypes as *Tetracystis vinatzeri* OTU_0084, and *Pseudostichococcus monallantoides* OTU_0155 have also been recovered from an airborne snow sample, and tree bark (Tesson and Šantl-Temkiv, 2018; sequence accession no. ON119327). The OTU_0121 *Coccomyxa subellipsoidea* genotype has been reported several times from Antarctica and recovered from tree bark in Germany (sequence accession no. ON119345). The OTU_0008 *Chamaetrichon basiliensis* found at the *Meseta* as well as at *SchF* shared full sequence identity with an environmental clone from soils in England (albeit misidentified as “Diptera”; Malik et al., 2018). Its next closest reference (with 2 sequence positions different), culture strain CCALA 986, has been isolated from the littoral zone of shallow lakes of James Ross Island at Antarctic Peninsula (Škaloud et al., 2013). It shares ITS2 sequence identity with culture strain SAG 2396, isolated from a freshwater creek in Germany. ITS2 genotypic identity with references from Germany was also found for *Klebsormidium* by the clone library approach of our study. Several of the 41 OTUs entire (genotypic) sequence identity to references were lichen photobionts, and two were snow algae (*Raphidonema catena, R. sempervirens*) also found in other cold geographic regions (Vančurová et al., 2018; Kim et al., 2020; Pröschold and Darienko, 2020; Yakimovich et al., 2021).

Another 66 *Meseta* algal OTUs exhibited high similarities to available references, i.e., 1.75 ≤ *NB* < 1.81 (Table 1), so they can still be regarded as the same species. Out of those only one OTU represented a species so far known only from Antarctica, i.e., *Coccomyxa antarctica.* It represents a photobiont of the lichen *Usnea aurantiaco-atra*, which covers the soil surfaces of Fildes Peninsula (Cao et al., 2018). The OTU_0011, identified as *Heterococcus virginis* (Xanthophyceae) was retrieved from all five *Meseta* study sites and the temperate site *SchF* with high read numbers. It shared a high ITS2 sequence similarity (*NB* = 1.76) with the authentic culture strain SAG 2163, defining the species. So far, the species was only known from the Maritime Antarctica (Rybalka et al., 2013), and our study revealed it to be distributed in a temperate site as well, i.e., *SchF*, for the first time.

Only eight algal genotypes found at the *Meseta* have been recorded from only the Polar regions so far (Table 1), and they may represent particularly cold-adapted specialist algae. Six specialist genotypes were from Antarctica, either in some proximity to Fildes Peninsula or at a far distance from it, Continental Antarctica. *Chloroidium antarcticum* and *Stichococcus antarcticus* are photobionts of pioneering lichens colonizing stony ground in the South Shetland Islands (Darienko et al., 2018; Beck et al., 2019). *Chlorominima collina*, the unidentified species of *Chloroidium* (Table 1), and *Raphidonema nivale* (OTU_0030) have been recovered from the colored snow of Livingston Island (Segawa et al., 2018), and King George Island (Gálvez et al., 2021; Yakimovich et al., 2021). In contrast, for *Chodatodesmus australis*, the reference strain is from Victoria Land (Andreoli et al., 1996), separated by an extended ice shield over a vast distance from Fildes Island. Similarly, the genotypic identity of algae of widely separated origins within Antarctica has already been reported for some members of Xanthophyceae (Rybalka et al., 2020). Those specialized indigenous algae may come from aeolian transport (Tesson et al., 2016) across ice shields from even harsher Antarctic areas. OTU_0979 *Protomonostroma dakshina* shared entire sequence identity with a sample of a newly described macroscopically large species of Ulvophyceae from a rocky intertidal marine habitat of East Antarctica (Kumari, Kaur, and Bast, pers. communication). Likely, that species may also be common on rocky shores of Maritime Antarctica, and fragments of that blade-forming alga may have been blown by wind from the seashore of Fildes Peninsula to the *Meseta*. *Coenochloris* sp. OTU_0923 had maximal sequence identity (*NB* = 1.81) with two references recovered only from the Arctic (as OTU-133 in Segawa et al., 2018, and as *Gloeocystis* sp. strain CCCryo142-0). Thus, our study revealed the genotype is distributed in both polar regions. Also, the OTU_0291 *Coleochlamys apoda* shared maximal sequence identity (*NB* = 1.81) with the epitype reference strain (CAUP H 7402-CRYO) defining the species (Barcytė et al., 2021). That strain has been isolated from the Arctic. Our study recovered the same genotype at the *Meseta*, as well as from the temperate reference site *SchF* (Supplementary Table 2). It suggests its wider distribution and that it is likely mesophilic rather than a cold-adapted specialist. There were another 30 OTUs that lacked any close reference sequences (*NB* < 1.75). Nevertheless, their distribution beyond Antarctica could be uncovered because they were within the overlap between the *Meseta* and the temperate site *SchF* (Supplementary Table 3).

With regard to the identified portion of 115 OTUs from the four targeted algal classes, our study favors the principle of microbial dispersal as suggested by Herbold et al. (2014) for Antarctic terrestrial microalgae. That principle concludes with the importance of aeolian transport in global-scale dispersal, which plays a significant role in the assembly of microbial communities over geological time periods. Air currents and migrating birds within Antarctica may be effective drivers for microalgae dispersal (Broady and Smith, 1994; Convey, 2010). The presence of specialist taxa suggests a unique adaptation to the particular combination of environmental conditions. They have the ability to outcompete exogenous microalgae under those environmental conditions (Herbold et al., 2014). Many species our study identified at the *Meseta* may have developed from the continuous immigration of viable algal propagules from more northerly landmasses via long-distance dispersal, e.g., as aeroplankton over the Southern Ocean (Smith, 1991). Due to the connectivity between the maritime Antarctic Region and temperate latitudes, there may be a high level of airborne immigration from exogenous sources (Kennedy, 1996). The microscopic soil algae, adapted to drought and high UV/PAR radiation, may easily be distributed worldwide and thus have colonized Antarctica many times after glaciation. It favors the external origins of microscopic soil algae, e.g., Fell et al. (2006) over the ‘glacial refugia hypothesis’ (Boenigk et al., 2006; Convey et al., 2008; De Wever et al., 2009). Ongoing climate change likely favors species of potentially ubiquitous distribution (Kleinteich et al., 2017). Many strains in far southerly soil propagule banks cannot manifest themselves because of the short growing season, low temperatures, and other inhibitory factors. However, climate warming may re-activate the dormant soil microalgal flora, increasing soil microorganism diversity (Davey, 1991; Wynn-Williams, 1996a,b). Also, the steady increase of human activities in Antarctica poses another potential factor for introducing increasingly non-specialist algae. Over time, those exogenous algae may outcompete the specialized indigenous species.

Our findings suggest that geographical boundaries do not limit soil algae dispersal. Instead, environmental conditions lead to their distribution, following the traditional ubiquity hypothesis [Baas-Becking 1934, cited in De Wit and Bouvier (2006)]. In contrast, due to dispersal barriers, endemism has been suggested for diatoms abundant in Antarctic aquatic environments and some Antarctic aquatic coccoid green algae (De Wever et al., 2009; Kociolek et al., 2017; Verleyen et al., 2021). The severity of environmental conditions may impose considerable barriers on aquatic algae. However, the phylogenetic distinctness of Antarctic diatom lineages from their temperate counterparts still needs to be shown. Coccoid green algae from temperate aquatic environments still need to be better sampled to overcome the present limitations of taxonomic assignments of metabarcoding studies (Salmaso et al., 2022). This may explain the lack of sufficiently close references in the study of De Wever et al. (2009). Also, the conserved nature of 18S rRNA gene sequences makes them inappropriate for discriminating genotypes of confined geographical distribution.

### Colonization of the *Meseta* by soil algae

Mineral soils recently exposed to glacier retreat have proved valuable subjects for research into the primary colonization by bacteria, algae, and fungi (Mataloni et al., 2000; Bajerski and Wagner, 2013; Newsham et al., 2021). Microalgae, together with cyanobacteria, may take a leading role in the primary colonization of fellfield soils (Wynn-Williams, 1990, 1996a). Following the glacial retreat, algal communities would have developed from viable propagules deposited on newly exposed substrata (Broady, 1996). The algal propagules that arrive there may have been transported through wind or developed from the snow fields associated with glaciers. In the ice-free Antarctic terrestrial environment, the edaphic algae are concentrated in the top few centimeters of the soil profile and exposed to environmental and seasonal changes. All plots in our study in the area of the ice-free plateau *Meseta* were established along the entire mountain crest of Fildes Peninsula at comparable altitudes of approximately 110 m a.s.l., in similar inclination and microclimatic conditions (Boy et al., 2016). Consequently, the environmental conditions of soil algae differ from those of snow algae. In Maritime Antarctica, the snow fields are closely connected with the coastal regions and influenced by the marine realm, bird colonies, and higher human impact (Soto et al., 2020). In contrast, the algal communities in the ice-free surfaces of soils along the *Meseta* mountain crest plateau are dominated by temporal and cyclic changes due to the exposition to high wind currents from outside Fildes Peninsula, while winds from inside lead to erosion impacting the communities as well. The wind currents in several directions distribute the fine material produced by weathering with which microbiota are associated. These dynamics of environmental conditions, combined with diurnal freeze-thaw cycles, determine the distribution of microbiota along the mountain crest of Fildes Peninsula and can explain the high similarity in the soil algal community composition between the study plots.

While previous works pointed toward a relevant role for bacteria and fungi in colonizing soils under harsh environmental conditions and during succession, this was not found for algae. They were ubiquitous along the chronosequence (Fernández-Martínez et al., 2017). This could be due to the high capability of algae to adapt well to a broad range of harsh environmental conditions. Soil algae are less confined to certain environmental conditions than other soil microbiota. It will allow algae to adjust to all conditions along the *Meseta* chronosequence. Considering the age of deglaciation along the *Meseta* mountain crest (likely between 100 and 6,200 years; Boy, 2014), the colonization of the *Meseta* sites by soil algal species may have already stabilized a long time ago. Therefore, the minor differences in species composition and alpha diversity of the soil algal communities between the northern (younger) and southern (older) parts of the *Meseta* glacier retreat may be largely arbitrary. In much younger chronosequences (<100 years), bacteria and fungi were found to occupy dominant roles, and local factors (e.g., soil structure) have been found to affect the rate of microbial community assembly (Sigler et al., 2002; Fernández-Martínez et al., 2017; Garrido-Benavent et al., 2020). In contrast, even in the younger chronosequences, algae did not show clear successional patterns along the transect (Garrido-Benavent et al., 2020). However, in the harsh mineral soil sites of Maritime Antarctica, the composition of microalgal communities would also be more prone to modification due to the manifold local consequences of climatic change (González Garraza et al., 2011).

## Conclusion

The study has provided data that the eukaryotic algae at the surface of the fellfield soils of *Meseta*, a mountain crest of Fildes Peninsula in Maritime Antarctica, exhibit high biodiversity. However, our sequencing approaches were limited by the fact that just revealing the presence of certain genotypes does not necessarily indicate that the algae are present in metabolically active forms since they can remain dormant over extended periods under harsh environmental conditions. Given that limitation, future PCR-independent metagenomics or RNA-centered meta-transcriptomic studies (e.g., Urich et al., 2008) or those employing a sophisticated method of separating intracellular DNA (indicating intact and potentially viable cells) from extracellular DNA (mainly representing preserved DNA from dead cells; Schulze-Makuch et al., 2018), should reinvestigate the soil algal biodiversity in Antarctic soils. They should be complemented by cultures to obtain more references for the improved identification of Antarctic soil algae. For the first time, the soil algae from an area of the ice-free Maritime Antarctica, under hardly any influence by the marine realm and anthropogenic disturbances, have been studied using a molecular marker of high taxonomic resolution. The major part of the algal biodiversity, 685 of 830 (82.5%) OTUs, could not be identified to the species level due to insufficient representation in reference sequence databases. With respect to the small portion of *Meseta* algal OTUs, i.e., 115 or 13.9% of all algal OTUs, for which their distribution could be assessed, our findings indicate the composition of the fellfield soils of the *Meseta* of mostly typical soil algae that are not indigenous but also distributed outside the Polar regions. They may have originated from northern soil alga propagule banks dispersed over long distances across the Southern Ocean. Only a minor portion of the recovered diversity represented indigenous species from local sources, such as lichens covering the soil surfaces or adjacent snow fields. Changes in terrestrial ecosystem processes of the Antarctic Peninsula, induced by climate warming, strongly affect the soil microbiota, including algae (Yergeau et al., 2012; Kleinteich et al., 2017). Climate warming may have the potential of re-activating the dormant microalgal flora (Wynn-Williams, 1996b). Rapid responses of soil algae to soil warming experiments have been observed in some Antarctic soils (Wynn-Williams, 1996a). Those soil algae from exogenous sources may overgrow and outcompete the indigenous Antarctic soil microorganisms as a response to global warming (Yergeau et al., 2012). In such a scenario of changing climate, the invasion of transportation-resistant soil algae (e.g., those with dormant stages) to the openly exposed fellfield soils of Maritime Antarctica and their proliferation during Austral summer becomes more likely so that the abundance of widely-distributed soil algae will increase. In contrast, the specialized and indigenous algae arriving through aeolian transport (Tesson et al., 2016) across ice shields from the harsher inner Antarctic areas will decrease and be outcompeted by those of external origins (Kleinteich et al., 2017).

## Data availability statement

The datasets presented in this study can be found in online repositories. The names of the repository/repositories and accession number(s) can be found below: https://www.ncbi.nlm.nih.gov/, BioProject ID PRJNA681474. The sequences of the algal OTUs are available from the DDBJ/EMBL/GenBank databases under accession numbers OR065178 - OR066145 (see Supplementary Table 1), algal sequences from the clone library approach under the accession numbers OR101475 - OR101670 and OR149916 - OR149986 (see Supplementary Tables 1, 6).

## Author contributions

TF and NR conceived the research, secured the funding, and wrote the manuscript with input from all co-authors. AT and AN conducted the processing of the raw sequences. CR and NR generated the sequence libraries. NR conducted the cloning approach and all other laboratory work. MB and DN analyzed and visualized the data. JB, DB, and RG conducted the fieldwork in Antarctica. All authors contributed to the manuscript and approved the submitted version.
