# Supplementary material for: Unrecognized diversity and distribution of soil algae from Maritime Antarctica (Fildes Peninsula, King George Island)
Source: Front Microbiol. 2023 Jun 26;14:1118747. doi: 10.3389/fmicb.2023.1118747 (PMC10332270; doi:10.3389/fmicb.2023.1118747)
Supplement: Supplementary file 1 [file Data_Sheet_1.zip › Data Sheet 1.pdf]

Supplementary Material

Unrecognized diversity and distribution of soil algae from Maritime Antarctica (Fildes Peninsula, King George Island)

Nataliya Rybalka, Matthias Blanke, Ana Tzvetkova, Angela Noll, Christian Roos, Jens Boy, Diana Boy, Daniel Nimptsch, Roberto Godoy, Thomas Friedl*

*** Correspondence:** Corresponding Author: tfriedl@uni-goettingen.de

1 Supplementary Data

Supplementary Table S1 (Table_S1_Meseta_SchF_1003_OTUs_stats.xlsx)
List of all 1003 ITS2 OTUs, the assignment of the algal OTUs to class, their identity (*NB*, pairing significance) with the closest sequence reference, geographic origin (if OTU is listed in the **Supplementary Table S2**), distribution on the studied samples with read numbers, and information with respect to the cloning approach. Sequence accession numbers in brackets indicate those closest references that have been misidentified not as algae (e.g., uncultured fungus) in the Genbank database. The seven *Meseta* samples and the sample from site *SchF* correspond to the following Sequence Read Archive accession numbers of the NCBI BioProject ID PRJNA681474:
AM31-13, SAMN16954457
AM06-13, SAMN16954455
AM06-14, SAMN16954458
AM09-13, SAMN16954456
AM09-14, SAMN16954452
AS14-14, SAMN16954453
AS15-14, SAMN16954454
SchF, SAMN16954458.

Supplementary Table S2

The 49 algal OTUs from the *Meseta*, including 24 overlap OTUs from temperate reference site *SchF* as well, with entire sequence identity (*NB* = 1.81) with their closest references, species identifications, number of references (*n* > 1), sequence accession numbers (Genbank) of references, and geographic origins

Class	Species	OTU ID	Reference(s)[1,2]	Origin	SchF
Chlorophyceae (18)	***Chlorominima collina***	OTU_0209	4; LC381738, MW553075, HQ404890, HQ404887	Antarctica	
	Chlorosarcinopsis eremi	OTU_0197	2; MT340906, (OW834951)	China, Russia	TRUE
	Chodatodesmus australis	OTU_0458	AM419228	Antarctica	
	Coelastrella striolata	OTU_0047	8; e.g., (KY935339), MG523306, MZ920145, FR865601, MT896388	Japan, Micronesia, Russia	TRUE
	Coelastropsis costata	OTU_0148	MK975488	Europe (Bulgaria)	
	***Coenochloris* sp.**	OTU_0923	HQ404874	Arctic (Svalbard)	
	Coleochlamys apoda	OTU_0291	MW244671	Arctic (Svalbard)	TRUE
	Desmodesmus denticulatus	OTU_0783	MK975487	Europe (Germany)	TRUE
	Heterochlamydomonas sp.	OTU_0417	2; (KX195464, OW839947)	China, USA	TRUE
	Hormotilopsis gelatinosa	OTU_0126	4; (KX193672, OW841667, ON696504), HQ646380	Australia, China, USA	TRUE
	Spongiococcum aplanosporum	OTU_0383	2; MG523298, MF569469	USA, Russia	TRUE
	Tetracystis sarcinalis	OTU_0864	(OW840043)	China	TRUE

	*Tetracystis vinatzeri***	OTU_0084	3; (KX194705, OW838452), MK005097	China, USA	TRUE
	Tetradesmus obliquus	OTU_0523	51; e.g., KU291882, MF326557, MG022741, MN636314, MZ546607	e.g., China, Switzerland, Turkey, Europe (Germany, Spain, Sweden),	
	unident. Chlamydomonadales	OTU_0332	2; MF483734, (OW839885)	China, Europe (UK)	TRUE
	unident. chlorophyte	OTU_0190	(OW835373)	China	TRUE
		OTU_0306	MN903344	Europe (Lithuania)	TRUE
		OTU_0398	MF484096	Europe (UK)	TRUE
Trebouxio-phyceae (28)	*Apatococcus* sp.**	OTU_0640	4; MK005076, KX025114, KX025109, ON119418	airborne snow sample, Europe (Germany)	TRUE
	*Asterochloris pseudoirregularis**	OTU_0039	73; e.g., KP314537, MH415323, MN598844, MT036566, MW043580	e.g., Antarctica, Arctic (Svalbard), Canada, Europe (Austria, Finland, Switzerland), Iceland, Japan, Russia, USA (Alaska)	
	*Asterochloris stereocaulonicola**	OTU_0196	5; MT036570, MT036571, MT036569, MH415218, MH415290	Antarctica, Argentina	
	Chlorella vulgaris	OTU_0772	2; MF482255, KX094790	Europe (Germany, UK)	TRUE
		OTU_0827	63; e.g., KX094797, KY229195, MN248530, MZ546604, OQ208834	e.g., Antarctica, China, Europe (Germany, Finland, Sweden, UK), South Korea, USA	TRUE
	Chloroidium antarcticum*	OTU_0179	MH551519	Antarctica	
	*Chloroidium lichenum**	OTU_0655	MH415403	New Zealand	

***Chloroidium* sp.**	OTU_0800	LC381752	Antarctica	
*Coccomyxa subellipsoidea**	OTU_0121	9; e.g., AC277064, HG972972, MG696578, MN871439, ON119345	Antarctica, China, Europe (Czech Republic, Germany), USA, Russia	TRUE
Diplosphaera sp.	OTU_0187	(KX221870)	New Zealand	
Edaphochloris andreyevii	OTU_0080	(KX195090)	USA	
Elliptochloris sp.*	OTU_0641	MH258956	Korea	TRUE
	OTU_0231	(OW841133)	China	
Laetitia sardoa	OTU_0577	2; (KX195613, OU941034)	USA, Europe (Sweden)	TRUE
*Myrmecia pyriformis**	OTU_0005	5; (MG207170, OX032879, OW838985), FJ554300, MW471028	Canada, China, Europe (Austria, Germany), USA	
Parietochloris bilobata	OTU_0182	3; LC639357, ON696501, (OX033079)	Australia, Europe (Austria, Germany)	TRUE
Pseudostichococcus monallantoides	OTU_0155	3; LC381755, (OW834355), ON119327	Antarctica, China, Europe (Germany)	TRUE
	OTU_0168	7; e.g., MT078186, MT425967, ON119232, (ON963159), (OW840264)	USA, China, Europe (Germany, Lithuania), Russia	TRUE
Raphidonema catena	OTU_0337	5; MT261819, (OW840117, OU938825), MW077573, MW077570	Arctic (Svalbard), China	
Raphidonema nivale	OTU_0030	3; MW077564, MW077565, MW077567	Antarctica	

	Raphidonema sempervirens	OTU_0115	10; e.g., AB903011, MK262783, MK262783, MW077549, MW077575	Arctic (Svalbard), Canada, Iceland, New Zealand	
	Stichococcus antarcticus*	OTU_0249	MH681103	Antarctica	
	*Trebouxia suecica**	OTU_0013	>100; e.g., KX147257, KY066324, MN397111, MN412814, MN654632	e.g., Antarctica, Europe (Norway, Poland, Spain, Sweden), Iceland	
	*Trebouxia vagua**	OTU_0114	2; KR912844, KR912849	USA	
	unident. trebouxiophyte	OTU_0028	FJ552952	Canada	
		OTU_0157	(OW840573)	China	
		OTU_0184	3; (MF484292, KX222759), JX435333	Europe (UK, Switzerland), New Zealand	
		OTU_0953	(KM494297)	Sweden	
Ulvophyceae (3)	*Chamaetrichon basiliensis*	OTU_0008	(MF034631)	Europe (UK)	TRUE
	Planophila laetevirens	OTU_0001	(OX032980)	Europe (Germany)	TRUE
	Protomonostroma dakshina	OTU_0979	OP866268	Antarctica	

[1]If $n > 10$, then only a selection of accession numbers is shown
[2]Sequence accession numbers in brackets indicate those closest references that have been misidentified not as algae (e.g., uncultured fungus) in the Genbank database
*long-distance dispersal as a symbiont of a lichen propagule likely
** from airborne sample
bold, specialist species so far known only from Polar regions

Supplementary Table S3

The 30 algal OTUs of the overlap between *Meseta* and the temperate reference site *SchF* (see **Figure 3**) and had low identities (*NB* < 1.75) with their closest available references

**Class**	**Species**
Chlorophyceae (7)	*Chlamydomonas* sp. (OTU_0713), *Chlorococcum* sp. (OTU_0686), *Spongiococcum* sp. (2; OTU_0376, OTU_0377), unident. Chlamydomonadales (2; OTU_0323, OTU_0725), unident. chlorophyte (OTU_0792*)*
Trebouxiophyceae (4)	*Neocystis* sp. (2; OTU_0434, OTU_0860), unident. trebouxiophyte (2; OTU_0367, OTU_0585)
Ulvophyceae (7)	*Chamaetrichon* sp. (OTU_0154), *Planophila* sp. (6; OTU_0042, OTU_0050, OTU_0339, OTU_0390, OTU_0515, OTU_0887)
Xanthophyceae (12)	*Heterococcus* sp. (4; OTU_0051, OTU_0246, OTU_0272, OTU_0555), unidentified xanthophyte (8; OTU_0116, OTU_0129, OTU_0217, OTU_0564, OTU_0584, OTU_0653, OTU_0801, OTU_0815)

Supplementary Table S4

The 36 algal OTUs found in both the northern and the southern part of the *Meseta*, as well as the temperate reference site *SchF*, with respect to the four targeted algal classes and their similarities with reference sequences

**Class**	**Identity level**	**OTU**
Chlorophyceae	genotypes[1]	*Coelastrella striolata* OTU_0047, *Coleochlamys apoda* OTU_0291, unident. Chlamydomonadales OTU_0332, unident. chlorophyte OTU_0190
	species[2]	*Coelastrella* sp. OTU_0029, *Chlorococcum* sp. OTU_0134, OTU_0174
	NB < 1.75	*Chlorococcum* sp. OTU_0686
Trebouxiophyceae	genotypes[1]	*Chlorella vulgaris* OTU_0772, *Coccomyxa subellipsoidea* OTU_0121, *Laetitia sardoa* OTU_0577, *Parietochloris bilobata* OTU_0182, *Pseudostichococcus monallantoides* OTU_0155, OTU_0168
	species[2]	*Coccomyxa viridis* OTU_0215, *Elliptochloris subsphaerica* OTU_0235, *Elliptochloris* sp. OTU_0105, *Muriella terrestris* OTU_0349, *Myrmecia* sp. OTU_0101, *Neocystis mucosa* OTU_0069, unident. Chlorellales OTU_0538, unident. trebouxiophyte OTU_0258
	NB < 1.75	*Neocystis* sp. OTU_0434
Ulvophyceae	genotypes[1]	*Chamaetrichon basiliensis* OTU_0008, *Planophila laetevirens* OTU_0001
	NB < 1.75	*Planophila* sp. OTU_0042, OTU_0050, OTU_0339, OTU_0390
Xanthophyceae	species[2]	*Heterococcus virginis* OTU_0011
	NB < 1.7	*Heterococcus* sp. OTU_0246, OTU_0051, unident. xanthophyte OTU_0116, OTU_0129, OTU_0584, OTU_0801

[1], entire sequence identity with reference (*NB* = 1.81)

[2], species level identity (1.75 ≤ *NB* < 1.81)

Supplementary Table S5

Distribution of the 830 *Meseta* algal OTUs on the two parts of the *Meseta*, as well as the temperate reference site *SchF*, with respect to the four targeted algal classes

	**OTUs in the overlap *Meseta* and *SchF***	**OTUs found in both parts of the *Meseta*, as well as *SchF***	**OTUs found in both the northern and the southern part of *Meseta***	**OTUs found in only the northern part of *Meseta***	**OTUs found only in southern part of *Meseta***
Chlorophyceae (68)	29 (42.6 %)	8 (11.8 %)	33 (48.5 %)	13 (19.1 %)	1 (1.5 %)
Trebouxiophyceae (363)	22 (6.1 %)	15 (4.1 %)	237 (65.3 %)	106 (29.2 %)	13 (3.6 %)
Ulvophyceae (251)	11 (4.4 %)	6 (2.4 %)	152 (60.6 %)	84 (33.5 %)	10 (4.0 %)
Xanthophyceae (148)	13 (8.8 %)	7 (4.7 %)	75 (50.7 %)	60 (40.5 %)	7 (4.7 %)
all *Meseta* algae (830)	**75 (9.0 %)**	**36 (4.3 %)**	**497 (59.9 %)**	**263 (31.7 %)**	**31 (3.7 %)**

Supplementary Table S6 (Table_S6_unmatched_clones.xlsx)

The 71 sequences from the clone library approach that revealed species not recovered by the paired-end approach, their identity (*NB*, pairing significance) with the closest reference sequence, and distribution on the studied sites

Supplementary File S1
Detailed description of the consensus approach used for assigning the taxonomic label for each OTU

The taxonomic label was chosen as the assigned consensus taxonomy of each OTU was determined using the bit score S’ of the first 10 hits of the BLASTN query (Altschul et al., 1997) using R (Version 4.0.2; R Core Team (2020) and the data.tree package (Glur, 2020). The latter create trees structures from hierarchical data. For each hit a path was defined as the consecutive order of its taxonomic ranks, i.e., kingdom, phylum, class, order, family, genus and species. An additional node ”Origin” was prepended to the path, and the bit score of the corresponding hit was stored as an attribute. Finally, a tree was constructed using the defined paths of the hits. Only unique paths were used for the tree construction. If there were duplicated paths, the bit score of the corresponding hits was summed up. Each node stores the cumulative bit score attribute. The ”Origin” node stores the cumulative bit score of all the first 10 hits, and each leave node only the bit score of the corresponding hit (or hits in the case of duplicated paths). All the nodes between the ”Origin” and the leave nodes store the cumulative bit score resulting from the paths that pass through them. The consensus taxonomy of each OTU is the path of the tree with the highest cumulative bit score. Alignment statistics used in later analyses are those of the hit, which path has the highest cumulative bit score. If several hits were represented by the determined consensus taxonomy, the alignment statistic of the first of these hits was used.

Altschul, S.F., Madden, T.L., Schäffer, A.A., Zhang, J., Zhang, Z., Miller, W., and Lipman, D.J. (1997). Gapped BLAST and PSI-BLAST: a new generation of protein database search programs. *Nucleic Acids Research* 25**,** 3389-3402. doi: 10.1093/nar/25.17.3389

Glur, C. (2020) data.tree (version 1.0.0). General Purpose Hierarchical Data Structure. Written and maintained by Christoph Glur <christoph.glur@ipub.com>, http://github.com/gluc/data.tree, https://CRAN.R-project.org/package=data.tree, https://rdocumentation.org/packages/data.tree/versions/1.0.0

R Core Team (2020). *R: A language and environment for statistical computing* [Online]. Vienna, Austria. Available: https://www.R-project.org/. [Accessed].

Supplementary Figure S1

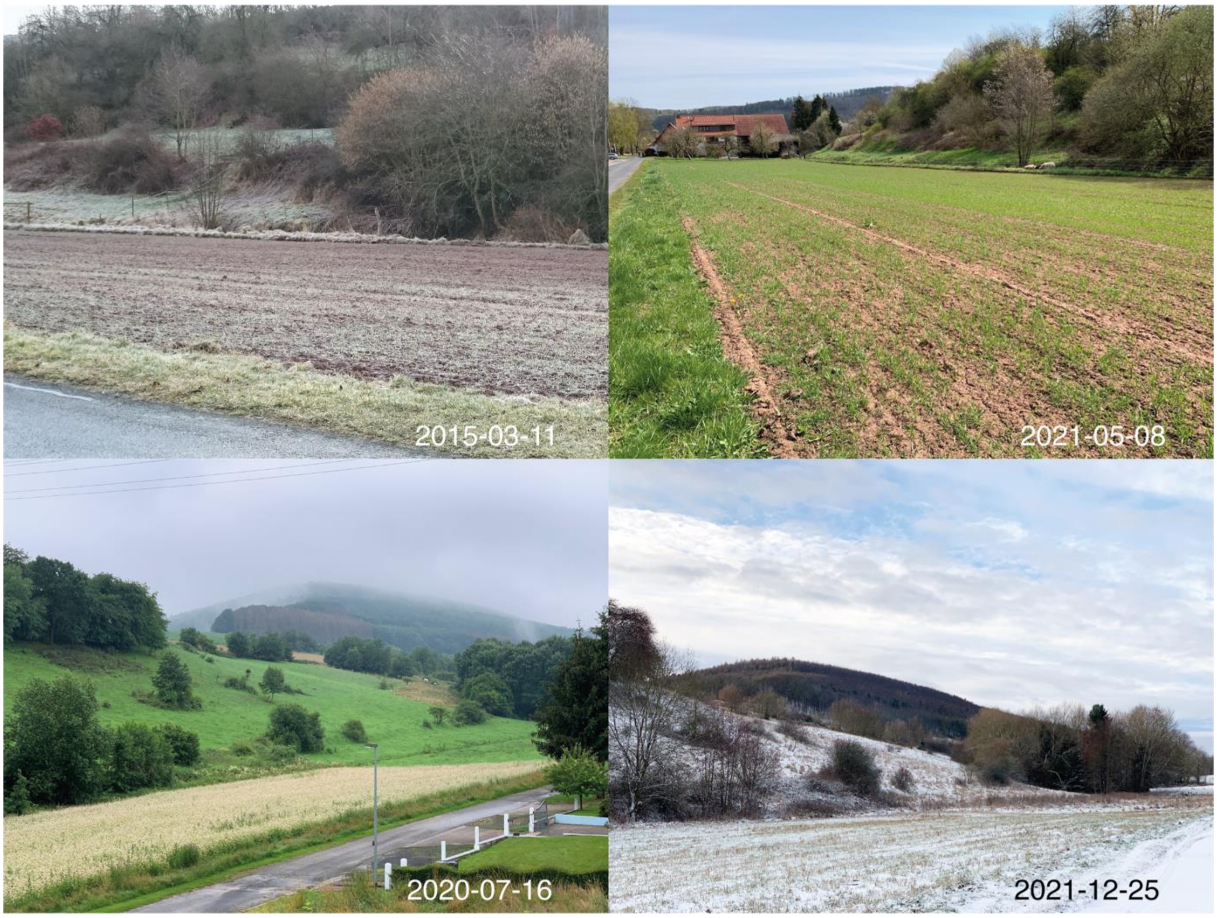

FIGURE S1 | Views of the temperate reference site *SchF* from three different years at different seasons. Upper left, the site at the time of collecting the soil surface subsamples before vegetation started. Site *SchF* represents a rural site in Germany under mild land use, i.e., without the usage of fertilizers and pesticides, to grow various forage crops which were changed yearly. It is located adjacent to the housing of the small village of Schlarpe, Uslar, in a valley of the Solling low mountain range (51.649111N, 9.750778E).
